# Impact of growth curve and dietary energy-to-protein ratio of broiler breeders on egg quality and egg composition

**DOI:** 10.1016/j.psj.2022.101946

**Published:** 2022-05-02

**Authors:** J. Heijmans, M. Duijster, W.J.J. Gerrits, B. Kemp, R.P. Kwakkel, H. van den Brand

**Affiliations:** ⁎ De Heus Animal Nutrition B.V., Ede 6717 VE, the Netherlands; † Animal Nutrition Group, Department of Animal Sciences, Wageningen University, PO Box 338, Wageningen NL-6700 AH, the Netherlands; ‡ Adaptation Physiology Group, Department of Animal Sciences, Wageningen University, PO Box 338, Wageningen NL-6700 AH, the Netherlands

**Keywords:** broiler breeder, feed strategy, modelling, egg components

## Abstract

Egg characteristics have an impact on embryonic development and post-hatch performance of broilers. The impact of growth curve (**GC**) and dietary energy-to-protein ratio of broiler breeder hens on egg characteristics was investigated. At hatch, 1,536 pullets were randomly allotted to 24 pens in a 2 × 4 factorial dose-response design with 2 GC (standard growth curve = SGC or elevated growth curve = EGC (+ 15%)) and 4 diets, differing in energy-to-protein ratio (defined as 96%, 100%, 104% and 108% AME_n_ diet). Feed allocation per treatment was adapted weekly to achieve the targeted GC and to achieve pair-gain of breeders within each GC. Breeders on an EGC produced larger eggs (∆ = 2.3 g; *P* < 0.001) compared to breeders on a SGC. An exponential regression curve, with age (wk) of the breeders, was fitted to describe the impact of GC and dietary energy-to-protein ratio on egg composition. Yolk weight was 0.8 g higher for eggs from EGC breeders than from SGC breeders ($a-108.1*0.907^{Age}$, where a was 22.1 and 22.9 for SGC and EGC, respectively; R^2^ = 0.97; *P*<0.001). An interaction between GC and dietary energy-to-protein ratio on albumen weight was observed (*P* = 0.04). Dietary energy-to-protein ratio did not affect albumen weight in SGC breeders ($42.7-56.2*0.934^{Age}$; R^2^ = 0.89), but for EGC breeders, a higher dietary energy-to-protein ratio resulted in a 0.9 g lower albumen weight from 96% AME_n_ to 108% AME_n_ ($a-62.9*0.926^{Age}$, where a was 43.4, 43.2, 42.8, and 42.5 for 96% AME_n_, 100% AME_n_, 104% AME_n_, and 108% AME_n_, respectively; R^2^ = 0.86). Albumen DM content decreased linearly with an increased dietary energy-to-protein ratio, but this was more profound in EGC breeders (β = −0.03 %/% AME_n_) than in SGC breeders (β = −0.01 %/% AME_n_; *P* = 0.03). Overall, it can be concluded that an EGC for breeders led to larger eggs with a more yolk and albumen, whereas dietary energy-to-protein ratio had minor effects on egg composition.

## INTRODUCTION

The hatch of healthy viable day-old chicks is crucial for health, welfare, and performance of broilers (Tona et al., 2005; Van de Ven et al., 2012). Day-old chick quality depends, among others, on the amount and quality of nutrients stored within the egg (Koppenol et al., 2015; Willems et al., 2015b; Iqbal et al., 2017), the ability of the embryo to use these nutrients (Yalçin et al., 2008), on albumen (Benton and Brake, 1996) and shell quality (Maina, 2017), and epigenetic factors (Lesuisse et al., 2018).

A fresh hatching egg contains approximately 50% protein, 40 to 43% lipids and 6% carbohydrates on a DM basis (Nangsuay et al., 2013, 2015). These egg nutrients are used by the embryo to develop. The yolk is a major energy source and both the yolk and the albumen are major protein sources for tissue synthesis in the developing embryo (Noble and Cocchi, 1990; Willems et al., 2014a). The shell controls the exchange of water and gasses through the pores in the shell and serves as a calcium source (Nys et al., 1999; Hincke et al., 2012). Variation in nutrient density, in the total amount of nutrients deposited in either of these components, or shell properties might therefore influence day-old chick quality (Lourens et al., 2006; Nangsuay et al., 2011, 2015).

Nutrients deposited within the egg are fixed at the moment of oviposition and should therefore contain all nutrients for the embryo to develop. Nutrients deposited in the egg originate either from mobilized body reserves of the breeder or from her diet (Ekmay et al., 2014; Salas et al., 2017). A change in breeder body reserves or diet composition might therefore influence nutrient deposition in the egg.

Total body reserves of the breeder hen can be changed by altering the growth curve during rearing and production (Van der Klein et al., 2018; Heijmans et al., 2021). A 15 to 22% higher growth curve from 0 to 60 wk of age resulted in approximately 200 to 230 g more body fat at 55 to 60 wk of age (Van der Klein et al., 2018; Zuidhof, 2018; Heijmans et al., 2021) and approximately 65 g more breast filet, as an indicator for more body protein (Van der Klein et al., 2018). It can be hypothesized that more body reserves of the breeder hen is beneficial for egg production and egg composition (Ekmay et al., 2014; Salas et al., 2017). In breeders, no effect on egg composition was observed when breeders were 7.5% heavier during rearing alone, but had a similar BW and body composition during production (Van Emous et al., 2013, 2015a). It remains unclear whether a higher BW during the production phase affects egg composition. In layers, it was observed that 8% heavier layers produced 1.2 g heavier eggs with a 0.6 g heavier yolk and 0.6 g heavier albumen compared to lighter layers (Pérez-Bonilla et al., 2012). It is hypothesized that heavier broiler breeders will produced larger eggs with a larger yolk, which eventually will be beneficial for chick quality (Nangsuay et al., 2015).

Another strategy to change breeder body reserves, while maintaining a similar BW, is by altering the dietary energy-to-protein ratio. In broiler breeders, feeding diets with 25% lower dietary CP or 8% higher dietary energy concentration from 0 to 60 wk of age resulted in 5 to 11% more body fat at the same BW (Lesuisse et al., 2017; Zuidhof, 2018; Heijmans et al., 2021). Body fat is mobilized for yolk production (Salas et al., 2017) and consequently, it can be hypothesized that more body fat will be beneficial for yolk production and eventually chick quality (Nangsuay et al., 2015). However, it was observed in breeders that a higher dietary energy-to-protein ratio, by a reduction of 22 to 25% dietary CP concentration, did not affect yolk weight, albumen height or shell thickness (Lesuisse et al., 2017), but led to a 1.3 to 4.8 g lower albumen weight (Joseph et al., 2000; Lesuisse et al., 2017) and 3.4 to 4.0 g lower day-old chick weight (Lesuisse et al., 2017). This suggests that a reduction in dietary CP might not be beneficial for egg composition and chick quality. It remains unclear whether a higher dietary energy-to-protein ratio, by an increased dietary energy content, while maintaining a similar CP content, might affect yolk weight or density without penalizing albumen weight and egg quality.

The aim of the current study was to investigate the impact of growth curve and dietary energy-to-protein ratio of broiler breeders on egg quality and egg composition.

## MATERIALS AND METHODS

### Experimental Design

The experiment consisted of a 2 × 4 factorial dose-response design with 2 growth curves (**GC**) (standard growth curve = **SGC** or elevated growth curve = **EGC**) and 4 diets, differing in energy-to-protein ratio, by step-wise increase in energy content from 96 to 108% at a similar CP content (defined as 96, 100, 104 and 108% AME_n_ diet). A dose-response design was applied in order to estimate potential linear and quadratic contrasts for dietary energy-to-protein ratio over a larger range of dietary energy content. At the start of the experiment (d 0), 1,536 Ross 308 female broiler breeder pullets, originating from a 37 wk old grandparent flock (Aviagen-EPI, Roermond, The Netherlands) were randomly placed in 24 pens (64 pullets per pen). Treatments were randomly assigned within 3 blocks of 8 pens (n = 3 per treatment) and continued up to 60 wk of age. Feed allocation per diet was adapted weekly to achieve pair-gain of breeders within each GC. All experimental protocols were approved by the Central Commission on Animal Experimentation (The Hague, the Netherlands), approval number 2018.W-0023.001.

### Breeders, Housing, and Management

A detailed description of this experiment was reported by Heijmans et al. (2021). In brief, each pen consisted of a floor area (4.9 m^2^) with wood shavings as bedding and an elevated slatted floor (5.1 m^2^). On the elevated slatted area, a track feeding system was placed with a grill preventing rooster access. Feed was provided once per day. Drinking nipples were also placed on the elevated slatted floor and water was supplied ad libitum. Pullets were reared on a 8L:16D (10 lux) photoperiod and instantly photo-stimulated at 21 wk of age (11L:13D), with a gradual increase up to 23 wk of age (13L:11D). Laying nests were available to the breeders from 20 wk of age onward. At 20 wk of age, all pens were standardized to 45 breeders per pen (4.5 breeders per m^2^), closest to the average pen weight and four 20-wk old Ross 308 roosters were placed per pen. Roosters were fed a commercially available diet once a day in a rooster feeding pan. Height of the feeding pan was adjusted to prevent female access.

### Experimental Diets and Feed Allocation

Experimental diets were formulated isonitrogenous. Dietary AME_n_ levels were step-wise increased from 96% to 108% (96%, 100%, 104%, and 108%), where the 100% AME_n_ treatment was the AME_n_ recommended by the breeding company (Aviagen, 2016a). Dietary AME_n_ was increased by a higher inclusion of crude fat (soy oil and lard) and starch (maize starch), while decreasing inclusion of crude fiber (cellulose and finely ground oat hulls). The 96% and 108% AME_n_ diets were produced first. The intermediate diets (100 and 104% AME_n_) were produced by homogeneous mixing 96 and 108% AME_n_ diets in a 2:1 (100% AME_n_) or 1:2 (104% AME_n_) ratio. A detailed description of the diets was reported by Heijmans et al. (2021). Dietary ingredients, and calculated and analyzed nutrient content of the experimental diets is presented in Table 1. The weekly growth target of the SGC was according to the breeder recommendation (Aviagen, 2016b), whereas the EGC targeted a 15% higher weekly growth relative to the SGC throughout rearing and production. Daily feed allocation was calculated and adjusted weekly based on the desired GC. As starting point to achieve pair-gain of breeders, feed allocation of the SGC was according to breeder recommendation (Aviagen, 2016b) and feed allocation of the EGC was 15% higher, compared to the SGC. Hereafter, growth and egg production in the week prior were the directives for calculating the daily feed allocation. Within each GC, daily feed allocation was adjusted weekly based on dietary energy-to-protein ratio to achieve pair-gaining. As starting point to achieve pair-gain of breeders, feed allocation of the 100% AME_n_ was according to breeder recommendation (Aviagen, 2016b). Feed allocation of the other treatments (96, 104, and 108% AME_n_) was adjusted relatively to the 100% AME_n_ treatment to achieve a similar daily AME_n_ intake. Hereafter, growth and egg production in the week prior were the directives for calculating the daily feed allocation.

**Table 1:** Dietary ingredients, and calculated and analyzed nutrients of diets (g/kg, as-fed basis).

Item	Starter 1 (0–21 d)		Starter 2 (22–42 )		Grower (43–112 d)		Pre-breeder (113–160 d)		Breeder 1 (161–280 d)		Breeder 2 (281–420 d)	
Ingredient	96% AME_n_	108% AME_n_	96% AME_n_	108% AME_n_	96% AME_n_	108% AME_n_	96% AME_n_	108% AME_n_	96% AME_n_	108% AME_n_	96% AME_n_	108% AME_n_
Maize	450.0	450.0	500.0	500.0	400.0	400.0	500.0	500.0	440.0	440.0	460.0	460.0
Wheat	100.0	100.0	100.0	100.0	100.0	100.0	100.0	100.0	100.0	100.0	100.0	100.0
Soybean meal	240.9	245.1	141.3	146.3	76.1	80.7	48.9	52.8	149.8	152.5	130.5	133.4
Sunflower meal	50.0	50.0	90.0	90.0	150.0	150.0	165.0	165.0	80.0	80.0	90.0	90.0
Wheat middlings	-	-	-	-	100.0	100.0	25.0	25.0	-	-	-	-
Oat hulls (fine)	50.0	1.0	56.0	5.1	65.0	19.3	50.0	1.0	48.0	1.0	46.6	1.0
Cellulose	44.1	1.0	47.9	5.0	50.0	5.0	46.8	1.0	44.5	1.0	45.2	1.0
Soya oil	11.1	17.8	9.5	14.3	8.0	12.0	5.0	7.0	4.8	10.8	11.9	14.9
Lard	3.0	4.2	4.2	6.8	3.3	6.7	5.0	10.2	29.5	34.9	23.5	32.1
Maize starch	14.0	94.5	14.3	96.2	19.9	99.2	11.7	96.1	14.7	91.6	1.0	76.9
Chalk	13.9	14.1	13.8	13.9	13.3	13.4	-	-	-	-	-	-
Limestone (coarse)	-	-	-	-	-	-	24.5	24.6	71.0	71.1	73.4	73.5
Monocalcium phosphate	9.8	9.2	10.5	9.9	5.4	4.9	5.8	5.2	6.0	5.5	6.5	5.9
Sodium bicarbonate	3.3	3.3	3.3	3.3	2.5	2.5	3.3	3.3	2.7	2.7	3.0	2.9
Salt	1.8	1.8	1.7	1.7	2.2	2.2	1.5	1.5	2.1	2.1	2.0	2.0
L-Lysine	1.73	1.69	1.88	1.80	0.23	0.15	1.63	1.58	0.44	0.42	0.36	0.34
L-Threonine	0.68	0.68	0.54	0.54	-	-	0.49	0.48	0.57	0.58	0.54	0.55
DL-Methionine	2.34	2.34	1.71	1.71	0.65	0.65	1.13	1.13	1.73	1.77	1.59	1.62
Choline Chloride-50%	0.8	0.8	0.8	0.8	0.8	0.8	1.5	1.4	1.4	1.3	1.5	1.4
Xylanase	0.1	0.1	0.1	0.1	0.1	0.1	0.1	0.1	0.1	0.1	0.1	0.1
Phytase	0.05	0.05	0.05	0.05	0.05	0.05	0.05	0.05	0.05	0.05	0.05	0.05
Premix rearing^1^	2.5	2.5	2.5	2.5	2.5	2.5	-	-	-	-	-	-
Premix laying^2^	-	-	-	-	-	-	2.5	2.5	2.5	2.5	2.5	2.5
Calculated content^3^												
AME_n_ (kcal/kg)	2,570	2,890	2,570	2,890	2,545	2,865	2,640	2,970	2,735	3,080	2,735	3,080
Crude protein	175.1	175.0	143.7	143.6	136.5	136.5	123.0	122.5	138.5	137.7	135.2	134.3
Crude fat	41.5	49.0	42.0	49.0	40.0	47.0	38.8	45.7	60.0	71.1	61.6	72.8
Crude fibre	77.1	37.7	88.0	48.3	111.5	71.5	105.6	64.3	81.4	42.0	85.2	43.9
Starch	379.5	446.9	408.6	477.5	371.5	438.5	407.5	480.4	368.2	434.4	373.8	436.0
Starch:fat	9.1	9.1	9.7	9.7	9.3	9.3	10.5	10.5	6.1	6.1	6.1	6.0
Linoleic acid	18.0	21.0	18.0	20.3	17.0	19.0	16.3	17.4	16.8	20.0	20.0	22.0
Digestible lysine	9.0	9.0	7.0	7.0	4.8	4.8	5.1	5.1	5.9	5.9	5.5	5.5
Calcium	9.8	9.8	9.8	9.8	8.9	8.9	13.1	13.1	31.0	31.0	31.0	31.0
Retainable phosphorus	4.1	4.1	4.1	4.1	3.3	3.3	3.2	3.2	3.2	3.2	3.2	3.2
Analyzed content												
Crude protein^4^	170.2	172.9	145.1	148.0	133.0	135.1	129.6	127.4	145.2	142.2	139.9	135.1
Crude fat^4^	37.0	43.2	38.3	44.3	39.0	42.4	33.1	41.1	57.6	66.8	58.2	67.3
Starch	401.0	463.0	408.0	472.0	377.0	431.0	415.6	486.3	376.4	436.8	371.7	432.5

1 Provided per kg diet: Vitamin A 10,000 IU; Vitamin D_3_ 3,000 IU; Vitamin E 100 IU; Vitamin K 3.0 mg; Vitamin B_1_ 3.0 mg; Vitamin B_2_ 6.0 mg; Vitamin B_6_ 4.0 mg; Vitamin B_12_ 20 μg; Niacinamide 35 mg; D-pantothenic acid 15 mg; Folic acid 1.5 mg; Biotin 0.20 mg; Iron 40 mg; Copper 16 mg; Manganese 120 mg; Zinc 90 mg; Iodine 1.25 mg; Selenium 0.3 mg.

2 Provided per kg diet: Vitamin A 10,000 IU; Vitamin D_3_ 3,000 IU; Vitamin E 100 IU; Vitamin K 5.0 mg; Vitamin B_1_ 3.0 mg; Vitamin B_2_ 12.0 mg; Vitamin B_6_ 5.0 mg; Vitamin B_12_ 40 μg; Niacinamide 55 mg; D-pantothenic acid 15 mg; Folic acid 2.0 mg; Biotin 0.40 mg; Iron 50 mg; Copper 10 mg; Manganese 120 mg; Zinc 90 mg; Iodine 2.0 mg; Selenium 0.3 mg.

3 Calculated according to CVB (2012).

4 Analyzed values were within boundaries of the analytical error.

### Measurements

#### Egg Weight and Laying Rate

Eggs were collected and weighed daily per pen. Average egg weight of all eggs produced, excluding double yolked eggs, was calculated per pen per week. Laying rate was calculated as the total number of eggs produced divided by the number of breeders per pen per week, corrected for mortality.

#### Egg Quality

Egg quality was determined weekly from 25 to 28 wk of age. Hereafter, egg quality was determined every other week until 60 wk of age, with exception from 42 and 48 wk of age. At each age, 10 settable eggs per pen were randomly selected for analysis. Eggshell breaking strength was measured at the equator of each egg, using an eggshell tester (Futura, Löhne, Germany). Albumen height was measured at approximately 1 cm distance from the yolk, using an albumen height gauge (TSS, York, UK). Eggshell thickness without membranes was measured at three regions of the egg (blunt end, equator, and pointed end) of 3 eggs per pen, using an electronic micrometer (Helios Preisser, Gammertingen, Germany). Albumen height, breaking strength, and shell thickness were averaged per pen per age.

#### Fresh Egg Composition

Fresh egg composition was measured from the same eggs as used for egg quality analysis. Eggs were weighed individually and thereafter the yolk was separated from the albumen and weighed. Eggshells, including shell membranes were tissue cleaned, dried at 180°C for 20 minutes, and weighed. Albumen weight was calculated as the difference between egg weight and the sum of yolk weight and eggshell weight. Yolk weight, shell weight, and albumen weight were averaged per pen per age.

#### DM Analysis

At 26, 28, 33, 36, and 60 wk of age, yolk samples, used for fresh egg composition, were pooled in three samples per age per pen. At the same ages, including 46 wk of age, albumen samples, used for fresh egg composition, were pooled in 3 samples per age per pen. The yolk and albumen samples were stored at −20°C for further analysis. Samples were freeze dried and DM determined by the proximate method (AOAC, 1990). Yolk and albumen dry matter percentage were averaged per pen per age.

### Statistical Analysis

All data were analyzed, using the Restricted Maximum Likelihood variance component analysis procedure within a linear mixed model (Genstat 19th Edition, 2019). Pen was used as the experimental unit for all analyses. Means and model residuals were checked on homogeneity of variance prior to analyses. The model used was:

$$Y_{ijkl}=\;\mu +GC_{i}+Diet_{j}+GC_{i}\;x\;Diet_{j}+Age_{k}+GC_{i}x\;Age_{k}+Diet_{j}\;x\;Age_{k}+GC_{i}\;x\;Diet_{j}\;x\;Age_{k}+Block_{l}+e_{ijkl}$$

where Y_ijk_ is the dependent variable, µ is the overall mean, GC_i_ is the growth curve (i = SGC or EGC), Diet_j_ is the energy-to-protein ratio in the diet (j = 96%, 100%, 104% or 108% AME_n_), GC_i_ x Diet_j_ is the interaction between growth curve and diet, Age_k_ is age of the breeder flock (k = 22 to 60 wk of age), Block_l_ is the block (k = 1, 2 or 3), and e_ijkl_ is the residual error. Preliminary analysis showed that interactions between GC and Age, Diet and Age, and between GC, Diet and Age were not significant for any of the variables and consequently they were excluded from the model. Age was excluded from the model for egg weight and laying rate analysis, as these variables were analyzed per week. Fisher adjustments were used for multiple comparisons of factorial analysis.

Additionally, effects of Diet and Diet × GC interaction were analyzed as linear or quadratic contrasts. If linear effects of dietary energy-to-protein ratio were observed, also within GC, the slope (β) is presented in the result section. If quadratic effects of dietary energy-to-protein ratio, also within GC, were observed, the estimated AME_n_ percentage at which the dependent variable was at the maximum (concave quadratic relation) or minimum (convex quadratic relation) was calculated and presented in the result section. Data are presented as LS means ± SEM.

Additionally, to describe differences in egg composition over time, weight of the yolk, albumen and shell for each GC, diet, and diet × GC interaction in relation to breeder age were fitted, using the nonlinear regression procedure in Genstat, analogue to Nonis and Gous (2013), based on the following exponential regression curve:

$$Y=a+b*c^{Age}$$

where Y is either yolk, albumen or shell weight and a,b and c are the fitted coefficients for the exponential regression curve and Age is the age of the breeder hen in wk. First, the model was fitted as a single curve with the same coefficients for each GC or diet (model I). Next, the model was step-wise expanded with a separate constant coefficient (a; model II) for parallel lines, with a separate constant (a) plus linear (b; model III) coefficients for separate lines, or with all coefficients separate (model IV), for each GC x diet interaction. After each model fit, it was evaluated whether or not the model significantly improved, compared to the previous model. A significantly lower residual mean square error, a lower Bayesian Information Criterion (**BIC**), and a higher R^2^ indicated a better fit, compared to the previous model. The final model used (I to IV), was the model that significantly improved the fit compared to the previous model and no significant improvement of the fit was observed of the next model. Estimated coefficients and R^2^ of fitted models are presented. All statements of significance are based on testing at *P* ≤ 0.05.

## RESULTS

Results on nutrient intake, BW development, and productive performance, including settable egg production are presented elsewhere (Heijmans et al., 2021). No differences between treatments were observed on total settable egg production. On average, settable egg production was 181.9 eggs per breeder from 22 to 60 wk of age.

### Laying Rate

**Figure 1:**
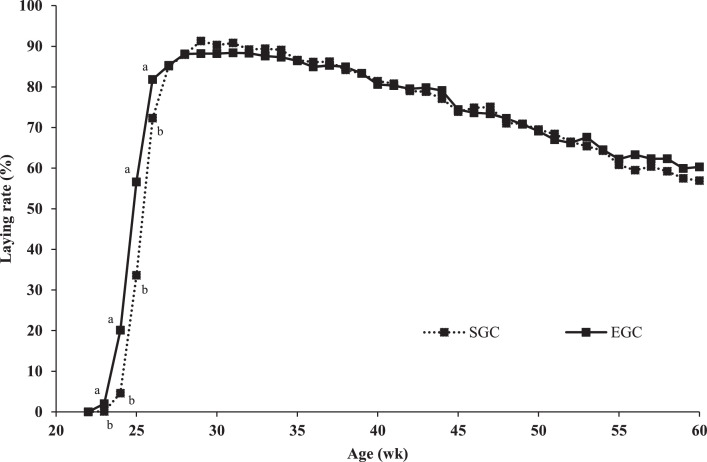
Laying rate of broiler breeders fed on 2 different growth curves (SGC = standard growth curve or EGC = elevated growth curve (+15%)) from 0 to 60 wk of age. ^a,b^LSmeans within age lacking a common superscript differ (*P* ≤ 0.05).

**Figure 2:**
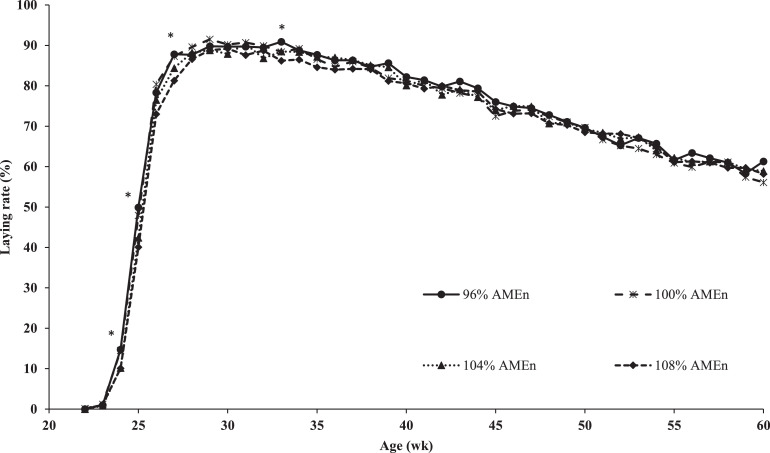
Laying rate of broiler breeders fed 4 diets, differing in energy-to-protein ratio (96, 100, 104, or 108% AME_n_), fed from 0 to 60 wk of age. *LSmeans within age with asterisk show a significant linear effect of energy-to-protein ratio (*P* ≤ 0.05).

An interaction between GC and dietary energy-to-protein ratio on laying rate at 28, 29, and 41 wk of age was observed (data not presented). At these ages, laying rate decreased linearly with an increasing dietary energy-to-protein ratio within EGC breeders (β = −0.5 %/% AME_n_ on average), whereas laying rate increased linearly with an increasing dietary energy-to-protein ratio within SGC breeders (β = 0.3%/% AME_n_ on average). EGC breeders had a higher laying rate from 23 to 26 wk of age than SGC breeders (∆ = 12.5 % on average; Figure 1). From 30 to 60 wk of age, with exception of the interaction at 41 wk of age, no differences in laying rate between EGC and SGC breeders were observed (Figure 1). Laying rate decreased linearly with an increasing dietary energy-to-protein ratio at 24, 25, 27, and 33 wk of age (β = −0.6 %/% AME_n_ on average; Figure 2). No other difference in laying rate between different dietary energy-to-protein ratio was observed from 34 to 60 wk of age, with exception of the interaction at 41 wk of age (Figure 2).

### Egg Weight

**Figure 3:**
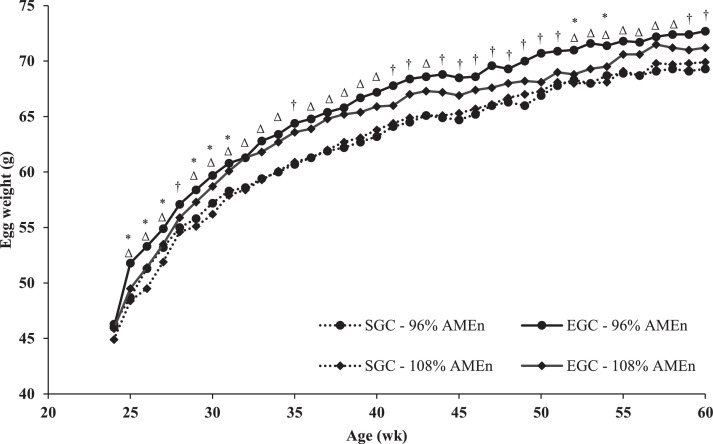
Egg weight of broiler breeders fed on 2 different growth curves (SGC = standard growth curve or EGC = elevated growth curve (+15%)) and 2 diets, differing in energy-to-protein ratio (96 or 108% AME_n_), from 0 to 60 wk of age. ^†∆*^LSmeans within age with a dagger (GC × diet (linear)), with a triangle (GC) or with an asterisk (diet (linear)) showed a significant effect (*P* ≤ 0.05). Data on the 2 intermediate diets (100% or 104% AME_n_) is not presented, as effects of dietary energy-to-protein ratio were linearly.

Egg weight was affected linearly by dietary energy-to-protein ratio. Therefore, only egg weights of the following treatments are presented; 96% AME_n_ SGC, 108% AME_n_ SGC, 96% AME_n_ EGC, and 108% AME_n_ EGC (Figure 3). An interaction between GC and dietary energy-to-protein ratio (linear) on egg weight was observed at 28, 35, 41, 42, 44 to 51, 59, and 60 wk of age (*P* ≤ 0.05; Figure 3). At all these ages, with exception of 28 wk of age, egg weight decreased linearly with an increasing dietary energy-to-protein ratio for EGC breeders (β = −0.13 g/% AME_n_ on average), whereas egg weight increased linearly with an increasing dietary energy-to-protein ratio for SGC breeders (β = 0.04 g/% AME_n_ on average). At 28 wk of age, in both GC, egg weight decreased with an increasing dietary energy-to-protein ratio, but this was more profound in EGC breeders (β = −0.12 g/% AME_n_) than in SGC breeders (β = −0.04 g/% AME_n_). Regardless of the interactions indicated above, at all ages EGC breeders produced heavier eggs than SGC breeders (∆ = 2.3 g on average; *P* < 0.001). At 25 to 31, 52, and 54 wk of age, a linear effect of dietary energy-to-protein ratio was observed (*P* < 0.05). Breeders with a higher dietary energy-to-protein ratio produced lighter eggs (β = −0.10 g/% AME_n_).

### Egg Quality

In total, egg quality of 4,320 eggs was determined over a period of 25 to 60 wk of age. In the first phase of lay (24–40 wk of age), no interaction between GC and dietary energy-to-protein ratio and neither an effect of dietary energy-to-protein ratio on albumen height was observed (Table 2). Eggs from EGC breeders had a lower albumen height than eggs from SGC breeders in this phase (∆ = 0.1 mm; *P* = 0.03). In the second phase of lay (41–60 wk of age) and over the total laying period, albumen height was 0.5 mm and 0.3 mm higher, respectively, in SGC breeders than in EGC fed at 96% AME_n_. This difference disappeared with a higher dietary energy-to-protein ratio in a quadratic way (*P* < 0.05).

**Table 2:** Average albumen height, breaking strength, and shell thickness during first phase of lay (22–40 wk), second phase of lay (41–60 wk) and the whole laying phase (22–60 wk) of eggs produced by broiler breeders fed on 2 different growth curves (SGC = standard growth curve or EGC = elevated growth curve [+15%]) and 4 diets, differing in energy-to-protein ratio (96, 100, 104, or 108% AME_n_), from 0 to 60 wk of age.

		22–40 wk			41–60 wk			22–60 wk		
Item		Albumen height (mm)	Breaking strength (N)	Shell thickness (µm)	Albumen height (mm)	Breaking strength (N)	Shell thickness (µm)	Albumen height (mm)	Breaking strength (N)	Shell thickness (µm)
Growth curve (n = 12)										
SGC		7.7^a^	38.1	363	6.8	38.2^a^	369	7.3	38.1^a^	366
EGC		7.6^b^	37.3	363	6.7	37.5^b^	367	7.2	37.4^b^	365
SEM		0.0	0.2	1	0.0	0.2	1	0.0	0.1	1
Diet (n = 6)										
96% AME_n_		7.7	37.6	363	6.8	37.7	366	7.3	37.6	364
100% AME_n_		7.6	37.9	361	6.8	38.0	371	7.2	38.0	365
104% AME_n_		7.7	37.7	364	6.7	37.6	367	7.2	37.6	365
108% AME_n_		7.7	37.6	364	6.7	38.2	368	7.2	37.8	366
SEM		0.0	0.3	2	0.1	0.3	2	0.0	0.2	1
Treatment (n = 3)										
SGC	96% AME_n_	7.7	37.9^ab^	364	7.0^a^	38.2	369	7.4^a^	38.0	366
	100% AME_n_	7.6	38.7^a^	363	6.7bcd	37.8	369	7.2^b^	38.3	366
	104% AME_n_	7.8	38.5^a^	363	6.8abc	38.0	368	7.3^a^	38.3	365
	108% AME_n_	7.8	37.3^b^	362	6.8abc	38.9	368	7.3^a^	38.0	365
EGC	96% AME_n_	7.6	37.2^b^	361	6.5^d^	37.3	364	7.1^b^	37.3	362
	100% AME_n_	7.6	37.1^b^	359	6.9^ab^	38.3	372	7.3^ab^	37.6	365
	104% AME_n_	7.6	37.0^b^	365	6.6^cd^	37.1	365	7.2^b^	37.0	365
	108% AME_n_	7.6	37.8^ab^	366	6.6^cd^	37.5	367	7.2^b^	37.7	367
	SEM	0.1	0.4	3	0.1	0.5	3	0.1	0.3	2
*P*-value										
Growth curve (GC)		0.03	0.003	0.90	0.003	0.05	0.53	<0.001	<0.001	0.71
Diet (factorial)		0.45	0.74	0.74	0.76	0.55	0.47	0.80	0.64	0.87
Diet (linear)		0.80	0.89	0.40	0.39	0.55	0.95	0.67	0.75	0.48
Diet (quadratic)		0.31	0.33	0.65	0.93	0.60	0.51	0.43	0.75	0.94
GC × Diet (factorial)		0.39	0.02	0.47	0.001	0.21	0.49	0.004	0.49	0.61
GC × Diet (linear)		0.29	0.11	0.16	0.26	0.29	0.78	0.96	0.71	0.21
GC × Diet (quadratic)		0.55	0.006	0.71	0.006	0.16	0.42	0.02	0.35	0.82
Age		<0.001	<0.001	<0.001	<0.001	0.003	<0.001	<0.001	<0.001	<0.001

a-d LSmeans within a column and factor lacking a common superscript differ (*P* ≤ 0.05).

In the first phase of lay (24–40 wk of age), a quadratic interaction between GC and dietary energy-to-protein ratio on breaking strength was observed (Table 2). Within the SGC, the highest breaking strength was estimated at 101% AME_n_ (∆_max_ = 1.5 N), whereas within the EGC, the lowest breaking strength was estimated at 101% AME_n_ (∆_max_ = −0.8 N). In the second phase of lay (41–60 wk of age) and over the total laying period (24–60 wk of age), no interaction between GC and dietary energy-to-protein nor a dietary energy-to-protein ratio effect on breaking strength was observed. In the second phase of lay (41–60 wk of age; ∆ = 0.7 N; *P* = 0.05) and over the total laying period (24–60 wk of age; ∆ = 0.7 N; *P* < 0.001), breaking strength was higher in eggs of SGC breeders than in eggs of EGC breeders. After correction for egg weight differences, differences in breaking strength were still significant between eggs from SGC and EGC breeders. No effect of GC, dietary energy-to-protein ratio, or the interaction between them, on shell thickness was observed (Table 2).

### Egg Composition

In total, egg composition of 4,320 eggs was determined over a period of 25 to 60 wk of age. Egg composition of the treatments during the first phase of lay (24–40 wk of age), second phase of lay (41–60 wk of age) and over the total laying period (24–60 wk of age) can be found in supplementary Table S1.

**Figure 4:**
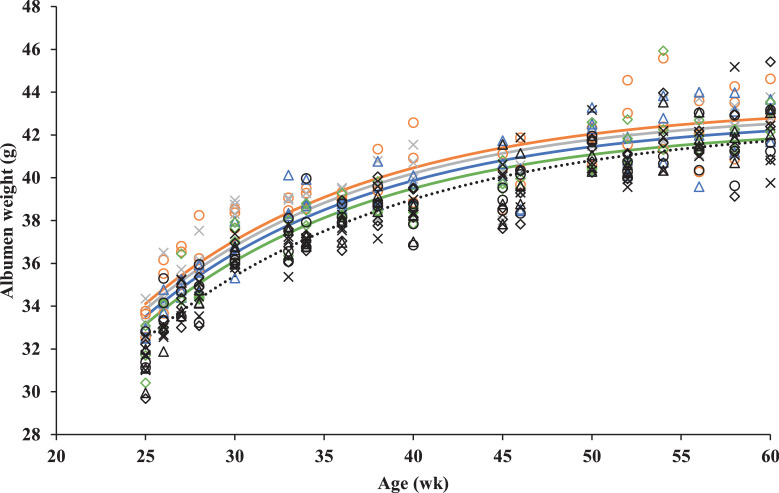
Observed (symbols) and predicted (lines) albumen weight of broiler breeders fed on a standard growth curve (black symbols, dashed line) and 4 diets, differing in energy-to-protein ratio; 96% AME_n_ (○), 100% AME_n_ (×), 104% AME_n_ (∆), or 108% AME_n_ (◊) or fed on an elevated growth curve (+15% compared to standard, solid lines) and 4 diets, differing in energy-to-protein ratio; 96% AME_n_ (red ○), 100% AME_n_ (gray ×), 104% AME_n_ (blue ∆), or 108% AME_n_ (green ◊), from 0 to 60 wk of age. Each symbol represents 1 replicate at each time point.

The exponential regression curves were fitted to describe the impact of GC and dietary energy-to-protein ratio on albumen, yolk and shell weight throughout the laying phase. An interaction between GC and dietary energy-to-protein ratio was observed on predicted albumen weight. In SGC breeders, dietary energy-to-protein ratio did not affect predicted albumen weight. A common line (model I) had the best fit (predicted albumen weight SGC = $42.7-56.2*0.934^{Age}$ (R^2^ = 0.89; *P* < 0.001)). However, in EGC breeders, the predicted albumen weight decreased in step-wise manner with 0.9 g when dietary energy-to-protein ratio increased from 96% AME_n_ to 108% AME_n_ (Figure 4; *P* < 0.001) Predicted albumen weight for EGC breeders could be expressed as $a-62.9*0.926^{Age}$ (R^2^ = 0.86; *P* < 0.001), where a was 43.4, 43.2, 42.8, and 42.5 for 96% AME_n_, 100% AME_n_, 104% AME_n_, and 108% AME_n_, respectively (*P* < 0.001). Regardless of the interaction indicated above, predicted albumen weight was always lower in SGC breeders than in EGC breeders (Figure 4).

**Figure 5:**
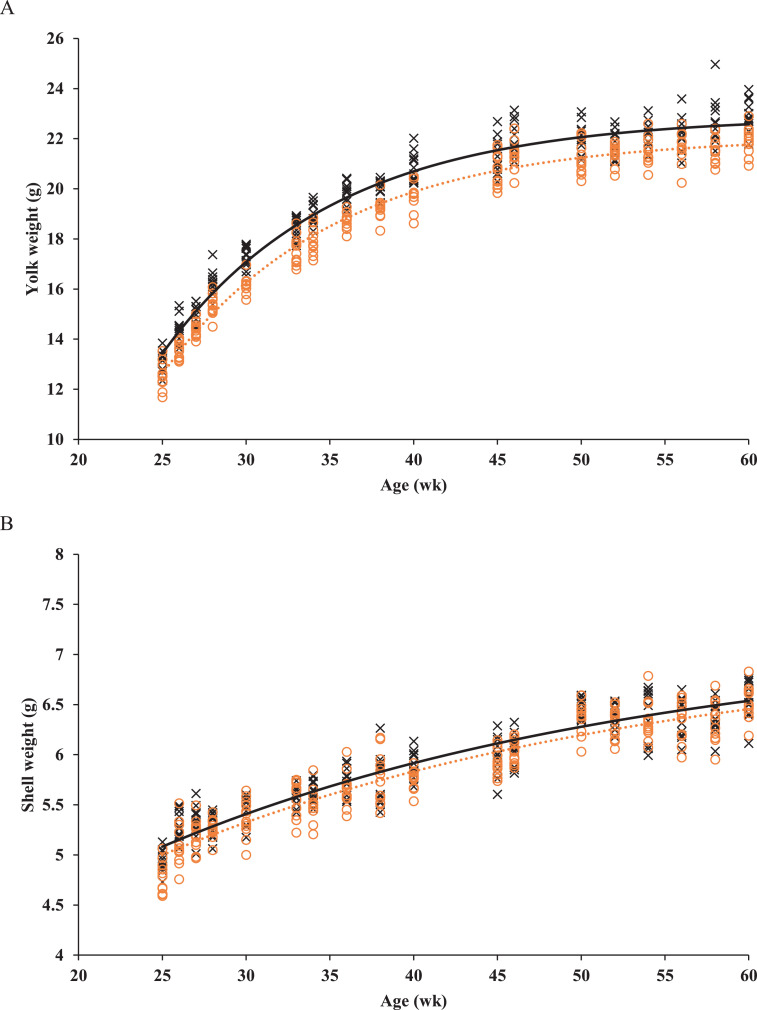
Observed (symbols) and predicted (lines) yolk weight (A) and shell weight (B) of broiler breeders fed on 2 different growth curves; standard growth curve (red ○, dashed line) or elevated growth curve (+15%; black ×, solid line) from 0 to 60 wk of age. Each symbol represents 1 replicate at each time point.

No interaction between GC and dietary energy-to-protein ratio or a dietary energy-to-protein ratio effect was observed on predicted yolk or shell weight (data not presented). Predicted yolk weight was 0.8 g higher for eggs from EGC breeders than from SGC breeders throughout the laying phase: predicted yolk weight = $a-108.1*0.907^{Age}$ (R^2^ = 0.97; *P* < 0.001), where a was 22.1 and 22.9 for SGC and EGC breeders, respectively (Figure 5). Predicted shell weight was 0.1 g higher for eggs from EGC breeders than from SGC breeders throughout the laying phase: predicted shell weight = $a-4.9*0.967^{Age}$ (R^2^ = 0.88; *P* < 0.001), where a was 7.1 and 7.2 for SGC and EGC breeders, respectively (Figure 5).

No effect of GC, dietary energy-to-protein ratio or the interaction between them was observed on DM content of the yolk (Table 3). A linear interaction between GC and dietary energy-to-protein ratio was observed on DM content of the albumen (Table 3). In both GC, a linear increase in dietary energy-to-protein ratio resulted in a linear decrease in DM content of the albumen, but this was more profound in EGC breeders (β = −0.03 %/% AME_n_) than in SGC breeders (β = −0.01%/% AME_n_; *P* = 0.03).

**Table 3:** Average egg yolk and albumen dry matter content of eggs produced by broiler breeders fed on 2 different growth curves (SGC = standard growth curve or EGC = elevated growth curve [+15%]) and 4 diets, differing in energy-to-protein ratio (96, 100, 104, or 108% AME_n_), from 0 to 60 wk of age.

Item		DM yolk^1^ (%)	DM albumen^2^ (%)
Growth curve (n = 12)			
SGC		50.9	13.9
EGC		51.0	13.9
SEM		0.1	0.0
Diet (n = 6)			
96% AME_n_		50.9	14.0
100% AME_n_		50.9	13.9
104% AME_n_		50.9	13.9
108% AME_n_		51.0	13.7
SEM		0.1	0.1
Treatment (n = 3)			
SGC	96% AME_n_	50.9	13.9bcd
	100% AME_n_	50.9	13.9abc
	104% AME_n_	50.9	14.0^ab^
	108% AME_n_	51.0	13.7^cd^
EGC	96% AME_n_	50.9	14.1^a^
	100% AME_n_	51.0	14.0^ab^
	104% AME_n_	51.0	13.8bcd
	108% AME_n_	50.9	13.7^d^
	SEM	0.1	0.1
*P*-value			
Growth curve (GC)		0.68	0.61
Diet (factorial)		0.87	0.001
Diet (linear)		0.40	<0.001
Diet (quadratic)		0.93	0.08
GC x Diet (factorial)		0.78	0.05
GC x Diet (linear)		0.65	0.03
GC × Diet (quadratic)		0.37	0.15
Age		<0.001	<0.001

a-d LSmeans within a column and factor lacking a common superscript differ (*P* ≤ 0.05).

1 Determined at 26, 28, 33, 36, and 60 wk of age.

2 Determined at 26, 28, 33, 36, 46, and 60 wk of age.

## DISCUSSION

The objective of this study was to evaluate effects of growth curve and dietary energy-to-protein ratio of broiler breeder hens on egg characteristics. Results will be discussed on main effects. Interactions will be discussed within the discussion of dietary energy-to-protein ratio.

### Growth Curve

In the current study, EGC breeders' cycle had on average a 12.5% higher laying rate in the first 4 wk of lay than SGC breeders. Sun and Coon (2005) and Van der Klein et al. (2018) also observed a 7.1 to 17.3% higher laying rate in the first 4 to 6 wk of lay for breeders that were 22 to 37% heavier at the end of rearing compared to standard breeders. The higher laying rate in the first weeks of the laying cycle can be explained by an earlier sexual maturation of heavier breeders (Sun and Coon, 2005; Renema et al., 2007; Van der Klein et al., 2018; Heijmans et al., 2021). From 30 wk of age onwards, no differences between GC in laying rate were observed, which is in line with Van der Klein et al. (2018). Currently, breeders are fed restrictedly to control their BW development in order to ensure good health and reproductive performance (Robinson et al., 1991; Bruggeman et al., 1999; Sun et al., 2006). In the current study and other studies, breeders with a 7.2 to 22.5% higher BW than standard (Van der Klein et al., 2018; Zukiwsky et al., 2021) or even ad libitum fed breeders (Zukiwsky et al., 2021) realized a similar rate of lay as breeders with a standard BW. All these results suggest that relaxation in feed restriction level might be possible, leading to an improved welfare of breeders, without negative effects on rate of lay. However, it remains unclear whether or not a higher than standard BW deteriorates fertility of breeders, which is another important factor for reproduction. Future studies should consider the impact of growth curve on fertility in current broiler breeders.

To our knowledge, only a limited number of studies are available on the impact of GC or BW of the broiler breeder hen (Van Emous et al., 2015a) or layer hen (Pérez-Bonilla et al., 2012) on egg quality parameters. Over the total laying period, some minor effects of GC on egg quality were observed, but it can be questioned whether or not these differences are relevant in perspective to offspring quality. Eggshell breaking strength was 0.8 N lower for eggs from EGC breeders than from SGC breeders. This was also observed after correction for differences in egg weight. Eggshell strength has been found to be positively related to the proportional eggshell weight and eggshell thickness, as reviewed by Roberts (2004). It was observed that eggshell thickness was similar between eggs from both GC, but as a proportion of egg weight, eggshells were smaller from EGC breeders than eggshells from SGC breeders, which might explain the lower eggshell breaking strength. A lower eggshell breaking strength in eggs obtained from EGC breeders, compared to SGC breeders might have negative effects on embryonic development, as (hairline) cracks lead to dehydration of the egg (Narushin and Romanov, 2002) during storage and incubation.

Albumen height, as a measure for albumen viscosity, was 0.1 mm lower in eggs from EGC breeders than from SGC breeders. Other studies in breeders (Van Emous et al., 2015a) and layers (Pérez-Bonilla et al., 2012) did not observe an effect of GC or BW on albumen height. Ovomucin is the main albumen protein responsible for albumen height (Silversides and Budgell, 2004; Wang et al., 2019), which might indicate a slightly lower deposition of albumen ovomucin in eggs from EGC breeders. A lower albumen viscosity might enhance oxygen transport to the embryo (Benton and Brake, 1996), leading to a higher hatchability and chick quality (Tona et al., 2003) for offspring from EGC breeders.

Eggs from EGC breeders were larger throughout the laying phase than eggs from SGC breeders. This has been previously discussed in Heijmans et al. (2021). These eggs from EGC breeders had a larger yolk, albumen, and shell, than eggs from SGC breeders. Predicted yolk weight showed parallel lines for GC in relation to breeder age. This means that the absolute difference in yolk weight between the GC remained similar throughout the laying phase, where the eggs from EGC breeders consistently had a 0.8 g larger predicted yolk. After correction for differences in egg weight between the GC, yolk was still relatively larger in eggs from EGC breeders. In layers, it was also observed that heavier layers produced larger eggs with a larger yolk compared to lighter layers (Pérez-Bonilla et al., 2012). We hypothesize that EGC breeders produce larger yolks due to their higher feed intake, more specifically due to their higher energy intake. Sun et al. (2006) observed higher plasma levels of insulin and triiodothyronine (**T_3_**) and a lower plasma level of glucagon with a higher feed intake. Higher plasma levels of insulin and T_3_ and lower glucagon levels stimulate de novo lipogenesis (Richards et al., 2003; Nguyen et al., 2008; Buyse and Decuypere, 2015). De novo lipogenesis synthesizes yolk precursors in the liver, like yolk directed very low density lipoproteins (**VLDL_y_**; Walzem et al., 1999; Buyse and Decuypere, 2015). These VLDL_y_ are transported to the ovary, where they are endocytosed in the yolk. The higher energy intake of EGC breeders might thus lead to a higher production of VLDL_y_, which results in larger yolks. In turn, it is expected that a larger yolk will be beneficial for chick quality (Nangsuay et al., 2015). Dry matter percentage of the yolk did not differ between the GC, indicating a similar total nutrient density for the yolk from both GC.

On average, a 0.9 g larger albumen and 0.1 g larger shell of eggs from EGC breeders seems consequential to a larger yolk. After ovulation, the yolk passes through the magnum, where the albumen is secreted around the yolk. A larger yolk might result in more distension of the lumen, which in combination with alterations in hormonal levels, induces signals to the storage granules of the albumen proteins to start secretion (Hiramoto et al., 1990; Johnson, 2015), finally resulting in a higher secretion of albumen proteins. Dry matter of the albumen did not differ between eggs from EGC or SGC breeders. A larger egg, due to a larger yolk and albumen, is expected to be beneficial for day-old chick quality (Ulmer-Franco et al., 2010; Nangsuay et al., 2011; Willems et al., 2015a).

### Dietary Energy-to-Protein Ratio

In the first 4 wk of the laying cycle, each percent decrease in dietary AME_n_ increased laying rate with 0.6% in both GC. The higher laying rate was probably due to a maximum 14.1% difference in CP intake in this phase, as energy intake was comparable for breeders on the different diets (Heijmans et al., 2021). Other authors also observed a 1.5 to 10% higher laying rate in the first 4 to 5 wk of the laying cycle for breeders with a 4 to 22.6% higher CP intake, compared to a control (Joseph et al., 2000; Van Emous et al., 2015b; Lesuisse et al., 2017), whereas differences in dietary energy intake did not affect laying rate up to 60 wk of age (Van Emous et al., 2015b). A higher laying rate is due to an earlier sexual maturation of breeders fed a lower dietary energy-to-protein ratio (β = 0.14 d/% AME_n_; Heijmans et al., 2021), which in turn is related to breeder body composition (Zuidhof, 2018; Salas et al., 2019; Hadinia et al., 2020). At 28 and 29 wk of age (peak production), a higher dietary energy-to-protein ratio increased laying rate in SGC breeders (β = 0.3 %/% AME_n_), whereas it decreased laying rate in EGC breeders (β = −0.5 %/% AME_n_). It can be speculated that total energy intake limited laying rate in SGC breeders. Another explanation might be that EGC breeders suffered from the relative high energy intake, leading to a fatty liver hemorrhagic syndrome, although in the current study incidence of fatty liver hemorrhagic syndrome was not determined. From 30 up to 60 wk of age, similar laying rates were observed regardless the dietary energy-to-protein ratio, which is comparable to results from Van Emous et al. (2015b). Other authors observed a 12% lower laying rate between 30 and 40 wk of age (Lesuisse et al., 2017) and 2.8% lower laying rate after 46 wk of age (Van Emous et al., 2018), when breeders were fed a diet with 12 to 25% lower CP compared to a control diet. For the period in between, 35 to 46 wk of age, they did not observe a difference in laying rate from 35 to 46 wk of age, when breeders were fed a 12 to 25% lower CP diet (Lesuisse et al., 2018; Van Emous et al., 2018). Combining results from all these studies suggests that laying rate is driven by dietary CP content rather than by dietary energy content, where a higher dietary CP content is beneficial for laying rate from start of production up to approximately 35 wk of age and after 45 wk of age. Between approximately 35 and 45 wk of age breeders mainly use body protein instead of dietary CP to support egg production (Ekmay et al., 2014; Vignale et al., 2017, 2018) and consequently dietary CP content is of less importance.

Over the whole laying period, dietary energy-to-protein ratio did not affect shell breaking strength and shell thickness. This is in line with results from Van Emous et al. (2015a) and Lesuisse et al. (2017). Some minor effects of dietary energy-to-protein ratio on albumen height were observed. However, differences were maximum 0.2 mm in albumen height and again, it can be questioned whether or not differences are relevant in perspective to offspring quality.

Egg weight was affected by dietary energy-to-protein ratio. From 25 to 31 wk of age, lowering dietary energy from 108 to 96% AME_n_ resulted in a linear increase of maximum 1.1 g in egg weight in both GC. The higher egg weight was probably due to a maximum 14.1% difference in CP intake, as energy intake was comparable for breeders on the different diets (Heijmans et al., 2021). This is in line with other authors, who observed a 0.8 to 5.8 g higher egg weight at comparable breeder ages, when CP intake was increased with 12.5 to 25% (Joseph et al., 2000; England et al., 2014; Lesuisse et al., 2017). At start of production, dietary CP is an important source for egg formation (Ekmay et al., 2014) and therefore an increase in CP intake might thus be beneficial for egg weight.

Later during production, from approximately 41 to 51 wk of age, a lower dietary energy-to-protein ratio was beneficial for egg weight for EGC breeders (β = −0.13 g/% AME_n_), whereas this was not observed in SGC breeders (β = 0.04 g/% AME_n_). The higher egg weight on a lower dietary energy-to-protein ratio for EGC breeders was almost entirely explained by larger (+0.9 g) albumen. Other authors also observed a 1.4 to 5.0 g higher egg weight, due to 1.3 to 4.8 g larger albumen, when breeders had a 22.6 to 25% higher CP intake (Joseph et al., 2000; Lesuisse et al., 2017). This might be explained by differences in CP availability during albumen synthesis. Albumen is synthesized and deposited in the magnum during a 3- to 4-h period when the yolk passes through the magnum (Hiramoto et al., 1990). A higher dietary CP availability in this period, when the yolk is in the magnum, increases synthesis of the albumen (Penz and Jensen, 1991). Although eating time was not determined for each treatment, visually it was observed that breeders with the lowest feed allocation (SGC 108% AME_n_) finished their daily portion around 4 to 6 h after feeding, whereas breeders with the highest feed allocation (EGC 96% AME_n_) finished their daily portion around 10 to 12 h after feeding. It can be speculated that EGC breeders still had feed (and thus dietary CP) available when the yolk passes through the magnum due to a higher feed allocation (Heijmans et al., 2021), whereas SGC breeders did not. A lower dietary energy-to-protein ratio thus led to a higher CP availability in EGC breeders, at the time the yolk is in the magnum, which in turn led to an increased deposition of albumen. In SGC breeders, dietary CP might not have been available any more at the moment the yolk is in the magnum, due to a lower feed allocation. Therefore, no effect of dietary energy-to-protein ratio on albumen weight was observed.

A lower dietary energy-to-protein ratio resulted in a higher DM percentage of the albumen (β = −0.02%/% AME_n_), although differences in DM percentages were maximal 0.4%. Albumen almost completely consists out of water and protein (Nangsuay et al., 2013). A maximum 14.1% higher CP intake for breeders on a lower dietary energy-to-protein ratio (Heijmans et al., 2021) might lead to a higher protein content of the albumen. Albumen is an important source of water and protein for tissue synthesis of the developing embryo (Willems et al., 2014a, 2015a,b; Da Silva et al., 2019). It has been observed that partial (3 mL) removal of albumen reduces prenatal protein availability and might have long-term negative consequences on performance and physiology of the offspring (Willems et al., 2014a,b, 2015a,b). It can thus be speculated that a 0.9 g higher albumen weight and a 0.4% higher DM in eggs from EGC breeders on a lower dietary energy-to-protein ratio (96% AME_n_), compared to a higher dietary energy-to-protein ratio (108% AME_n_), leads to a better offspring quality and performance.

Dietary energy-to-protein ratio had no effect on predicted yolk weight or yolk DM percentage. This was also observed by Peebles et al. (2000). Breeders on the different diets had a similar energy intake, but a linear decrease in CP intake with an increasing dietary energy-to-protein ratio (Heijmans et al., 2021). As discussed previously, energy intake might be the determinant for production of VLDL_y_ and ultimately yolk weight. Although no effects of dietary energy-to-protein ratio on albumen weight for SGC breeders or yolk weight for both GC were observed, it can be suggested that dietary energy-to-protein ratio might affect offspring quality and performance via potential epigenetic pathways (Lesuisse et al., 2017).

## CONCLUSIONS

It can be concluded that an elevated growth curve of broiler breeders or feeding a lower dietary energy-to-protein ratio led to a higher laying rate at start of production, potentially due to a higher CP intake or more CP in the body of the breeder hen. Growth curve or dietary energy-to-protein ratio had minor effects on egg quality. Breeders on an elevated growth curve produced larger eggs, with a more yolk, albumen and shell, compared to breeders on a standard growth curve, most probably due to a higher total nutrient intake. Dietary energy-to-protein ratio had minor effects on egg composition. Total energy intake of breeders might be the determinant for yolk weight. It is expected that a larger yolk and/or albumen will be beneficial for offspring performance. Future studies should consider the impact of growth curve and dietary energy-to-protein ratio on offspring quality and performance.

## Appendix Supplementary materials

Image, application 1

## Footnotes

Supplementary material associated with this article can be found, in the online version, at doi:10.1016/j.psj.2022.101946.
