# Supplementary material for: Impact of growth curve and dietary energy-to-protein ratio of broiler breeders on egg quality and egg composition
Source: Poult Sci. 2022 May 2;101(7):101946. doi: 10.1016/j.psj.2022.101946 (PMC9168161; doi:10.1016/j.psj.2022.101946)
Supplement: Supplementary file 1 [file mmc1.docx]

# SUPPLEMENTARY INFORMATION

**Table S1**. Average individual egg weight (EW), yolk, albumen, and shell expressed as percentage of the EW during first phase of lay (24-40 wk), second phase of lay (41-60 wk) and the total laying period (24-60 wk) of broiler breeders fed on 2 different growth curves (SGC = standard growth curve or EGC = elevated growth curve (+15%)) and 4 diets, differing in energy-to-protein ratio (96, 100, 104, or 108% AME_n_), from 0 to 60 wk of age.

|  |  |  | 24-40 wk | | | |  | 41-60 wk | | | |  | 24-60 wk | | | |
| --- | --- | --- | --- | --- | --- | --- | --- | --- | --- | --- | --- | --- | --- | --- | --- | --- |
| Item | |  | EW (g) | Yolk (%) | Albumen (%) | Shell (%) |  | EW (g) | Yolk (%) | Albumen (%) | Shell (%) |  | EW (g) | Yolk (%) | Albumen (%) | Shell (%) |
| Growth curve (n= 12) | | |  |  |  |  |  |  |  |  |  |  |  |  |  |  |
|  | SGC |  | 57.8 | 28.5 | 62.2 | 9.4 |  | 68.6^b^ | 31.2^b^ | 59.6 | 9.2^a^ |  | 62.6 | 29.7^b^ | 61.0 | 9.3 |
|  | EGC |  | 60.1 | 28.9 | 62.0 | 9.2 |  | 70.2^a^ | 31.6^a^ | 59.5 | 9.0^b^ |  | 64.6 | 30.1^a^ | 60.9 | 9.1 |
|  | SEM |  | 0.1 | 0.1 | 0.1 | 0.0 |  | 0.1 | 0.1 | 0.1 | 0.0 |  | 0.1 | 0.0 | 0.0 | 0.1 |
| Diet (n=6) | |  |  |  |  |  |  |  |  |  |  |  |  |  |  |  |
|  | 96% AME_n_ | | 59.3 | 28.5 | 62.3 | 9.2 |  | 69.6 | 31.5 | 59.6 | 9.0 |  | 63.9 | 29.8 | 61.1 | 9.1 |
|  | 100% AME_n_ | | 59.2 | 28.7 | 62.1 | 9.2 |  | 69.4 | 31.4 | 59.5 | 9.1 |  | 63.7 | 29.9 | 61.0 | 9.2 |
|  | 104% AME_n_ | | 58.7 | 28.7 | 62.0 | 9.3 |  | 69.4 | 31.4 | 59.5 | 9.1 |  | 63.4 | 29.9 | 60.9 | 9.2 |
|  | 108% AME_n_ | | 58.5 | 28.8 | 61.8 | 9.3 |  | 69.2 | 31.3 | 59.6 | 9.1 |  | 63.2 | 30.0 | 60.8 | 9.2 |
|  | SEM |  | 0.1 | 0.1 | 0.1 | 0.0 |  | 0.2 | 0.1 | 0.1 | 0.0 |  | 0.1 | 0.0 | 0.1 | 0.0 |
| Treatment (n=3) | | |  |  |  |  |  |  |  |  |  |  |  |  |  |  |
|  | SGC | 96% AME_n_ | 57.9^c^ | 28.3^c^ | 62.5^a^ | 9.3^b^ |  | 68.4 | 31.2 | 59.7 | 9.1 |  | 62.6^d^ | 29.6 | 61.2^a^ | 9.2^b^ |
|  |  | 100% AME_n_ | 57.9^c^ | 28.6^b^ | 62.0^bc^ | 9.3^b^ |  | 68.7 | 31.3 | 59.6 | 9.2 |  | 62.7^d^ | 29.8 | 61.0^b^ | 9.3^b^ |
|  |  | 104% AME_n_ | 57.6^c^ | 28.4^bc^ | 62.1^bc^ | 9.5^a^ |  | 68.8 | 31.3 | 59.5 | 9.2 |  | 62.6^d^ | 29.7 | 61.0^ab^ | 9.3^a^ |
|  |  | 108% AME_n_ | 57.6^c^ | 28.5^bc^ | 62.2^abc^ | 9.4^b^ |  | 68.5 | 31.2 | 59.7 | 9.1 |  | 62.5^d^ | 29.7 | 61.0^ab^ | 9.3^b^ |
|  | EGC | 96% AME_n_ | 60.7^a^ | 28.7^b^ | 62.2^abc^ | 9.2^c^ |  | 70.8 | 31.7 | 59.5 | 8.9 |  | 65.2^a^ | 30.0 | 61.0^ab^ | 9.1^c^ |
|  |  | 100% AME_n_ | 60.5^a^ | 28.7^b^ | 62.2^abc^ | 9.1^c^ |  | 70.1 | 31.6 | 59.4 | 9.0 |  | 64.8^ab^ | 30.0 | 61.0^ab^ | 9.1^c^ |
|  |  | 104% AME_n_ | 59.8^b^ | 29.0^a^ | 61.9^c^ | 9.1^c^ |  | 70.0 | 31.5 | 59.5 | 9.0 |  | 64.3^bc^ | 30.1 | 60.8^bc^ | 9.1^c^ |
|  |  | 108% AME_n_ | 59.3^b^ | 29.2^a^ | 61.5^d^ | 9.4^b^ |  | 69.8 | 31.5 | 59.5 | 9.1 |  | 64.0^c^ | 30.2 | 60.6^c^ | 9.2^b^ |
|  |  | SEM | 0.2 | 0.1 | 0.1 | 0.0 |  | 0.3 | 0.1 | 0.1 | 0.0 |  | 0.3 | 0.1 | 0.1 | 0.0 |
| P-value | |  |  |  |  |  |  |  |  |  |  |  |  |  |  |  |
|  | Growth curve (GC) | | <0.001 | <0.001 | 0.003 | <0.001 |  | <0.001 | 0.001 | 0.20 | <0.001 |  | <0.001 | <0.001 | 0.002 | <0.001 |
|  | Diet (factorial) | | <0.001 | 0.010 | <0.001 | 0.02 |  | 0.54 | 0.90 | 0.99 | 0.25 |  | <0.001 | 0.37 | 0.03 | 0.02 |
|  | Diet (linear) | | <0.001 | <0.001 | <0.001 | 0.006 |  | 0.19 | 0.47 | 0.91 | 0.11 |  | <0.001 | 0.08 | 0.002 | 0.002 |
|  | Diet (quadratic) | | 0.84 | 0.87 | 1.00 | 0.54 |  | 0.86 | 0.91 | 0.73 | 0.39 |  | 1.00 | 0.84 | 0.81 | 0.92 |
|  | GC x Diet (factorial) | | 0.04 | 0.007 | 0.002 | 0.02 |  | 0.13 | 0.78 | 0.93 | 0.29 |  | 0.009 | 0.10 | 0.04 | 0.008 |
|  | GC x Diet (linear) | | 0.004 | 0.04 | 0.04 | 0.51 |  | 0.06 | 0.33 | 0.86 | 0.16 |  | 0.002 | 0.39 | 0.17 | 0.16 |
|  | GC x Diet (quadratic) | | 0.75 | 0.08 | 0.003 | 0.005 |  | 0.18 | 0.69 | 0.74 | 0.72 |  | 0.21 | 0.09 | 0.02 | 0.02 |
|  | Age |  | <0.001 | <0.001 | <0.001 | <0.001 |  | <0.001 | <0.001 | 0.003 | <0.001 |  | <0.001 | <0.001 | <0.001 | <0.001 |

*^a-d^LSmeans within a column and factor lacking a common superscript differ (P≤0.05).*
